# Adolescents and health-related behaviour: using a framework to develop interventions to support positive behaviours

**DOI:** 10.1186/s40814-018-0259-7

**Published:** 2018-04-02

**Authors:** Jan Pringle, Lawrence Doi, Divya Jindal-Snape, Ruth Jepson, John McAteer

**Affiliations:** 1 Scottish Collaboration for Public Health Research and Policy, University of Edinburgh, 20 West Richmond St, Edinburgh, EH8 9DX UK; 2 Education, Inclusion and Life Transitions, University of Dundee, Dundee, UK

**Keywords:** Intervention development, Health behaviour, Adolescence, Intervention implementation

## Abstract

**Background:**

Experimentation is a natural part of adolescent maturation. In conjunction with increased exposure to behaviours such as alcohol or substance use, and the potentially intensified influence of peer groups, unhealthy behaviour patterns may develop as part of this experimentation. However, the adolescent years also provide considerable opportunity for behaviour to be shaped in positive ways that may improve immediate and longer term health outcomes. A systematic review carried out by the authors concluded that physiological changes during adolescence need to be considered when designing or implementing interventions, due to their influence on health behaviours. The aim of the study is to demonstrate how the six steps in quality intervention development (6SQuID) framework can be used, in conjunction with research or review findings, to inform the development of pilot or feasibility studies. Using the synthesised findings from our adolescent systematic review, we sought to illustrate how adolescent interventions might be designed to target specific health behaviours and augment positive adolescent health outcomes.

**Methods:**

We applied the 6SQuID framework to the findings from a review of adolescent physiological influences on health behaviour. This involved following the process defined within 6SQuID and applying the sequential steps to build a proposed pilot study, based on the pre-defined findings of our systematic review. We used the Social Learning Theory to assist in identifying how and why change can be influenced, with and for adolescents.

**Results:**

We devised a pilot study example, targeting teaching assistants, to illustrate how the detailed steps within the 6SQuID framework can assist the development and subsequent implementation of adolescent interventions that are likely to be effective.

**Conclusions:**

This paper gives details of how the 6SQuID framework can be used for intervention development, using specific research findings, across a variety of adolescent health behaviours. This example provides details of how to operationalise 6SQuID in practical terms that are transferrable to other populations and situations. In this respect, we anticipate that this illustrative case may be of use in the design, development, and implementation of a wide variety of interventions.

## Background

Adolescence is a process characterised by pubertal maturation, transition towards the mature social roles of adulthood, and the development of independence [1]. It is a time when new roles are acquired, personality becomes more individualised, and values are formed. Experimentation is a natural part of this process. In conjunction with increased exposure to behaviours such as alcohol or substance use, and the potentially intensified influence of peer groups, unhealthy behaviour patterns may develop as part of this experimentation. This may have consequences for both current and future health status [2]. However, the adolescent years also provide considerable opportunity for behaviour to be shaped in positive ways that may improve longer term health outcomes [2, 3]. Health interventions targeting this life stage therefore have the potential to promote positive outcomes during adolescence and beyond.

Recently, the authors conducted a systematic review relating to the physiological nature of adolescence and associated impacts on health behaviour [2]. We found that, due to their influence on health behaviour, physiological changes that occur during adolescence need to be considered when designing or implementing interventions to improve adolescent health outcomes. The review also further indicated that these changes should be viewed in the context of the wider social environment and not in isolation.

### The 6SQuID framework

The six steps in quality intervention development (6SQuID) framework provides a useful model to determine how best to develop interventions to maximise effectiveness. 6SQuID was developed due to a recognition that, whilst there was considerable literature relating to intervention evaluation, much less was available to guide development of public health interventions [4]. The framework supports better use of scarce public resources by ensuring that adequate attention is given to methodical intervention development, appropriate implementation, and thorough evaluation. Co-production with key stakeholders is central to the intervention development process outlined by the framework. The six steps detail a process that ensures due thought is given to the initial issues, by understanding health problems and their context, as well as those factors that have the greatest scope for change. 6SQuID builds upon existing intervention development frameworks, including the UK Medical Research Council’s guidance for developing and evaluating complex interventions [5], by providing a series of pragmatic steps (see Table 1). The framework is consistent with international guidance regarding the contribution that appropriate implementation can make to maximising the beneficial impact of health interventions [6].

**Table 1:** The 6SQuID process

6SQuID steps	Details
Step 1: define and understand the problem	Clarify the problem, using the existing research. Establish how the issues are socially and spatially situated, including any immediate or underlying influences. Diagrams may help at this point
Step 2: clarify which causal or contextual factors are malleable and have greatest scope for change	Identify the factors that shape the problem and have the greatest scope to be changed. Diagrammatic representation in step 1 may help to establish the most effective intervention point(s) in causal pathways
Step 3: identify how to bring about change: the change mechanism	Articulate the theory of change and mechanism(s) for incorporation into the intervention
Step 4: identify how to deliver the change mechanism	Investigate the means and options for delivering the intervention, as well as target groups and context
Step 5: test and refine on a small scale	Identify a means of testing the intervention in an appropriate setting, for a small sample of the target group(s), as detailed in step 4
Step 6: collect sufficient evidence of effectiveness to justify rigorous implementation or evaluation	Gather evidence that the intervention has worked as intended in the small scale, in order to warrant larger scale application. This may include critically examining any unintended/detrimental effects

Summarised from Wight et al. 2016

### Aims and objectives

We aimed to demonstrate how the 6SQuID framework [4] can be used, in conjunction with research or review findings, to inform the development of interventions. The synthesised findings from our adolescent systematic review were used to demonstrate ways in which potential adolescent interventions might be designed to target specific health behaviour determinants, in order to augment positive adolescent health outcomes.

## Methods

Our team (the authors) included three researchers involved in the synthesis of the systematic review findings (JMcA, JP, RJ), two researchers involved in 6SQuID development (LD, RJ), and one adolescent education and implementation expert (DJS). Taking the review findings, one author (JP) worked through the 6SQuID framework, applying and relating each step to the findings, and vice versa. These steps were critically reviewed by the other authors (JMcA, LD, RJ) and amendments made until agreement was reached. Final overview for consistency and relevance was then made (DJS). The detailed process and results are described below.

### Application of 6SQuID

We applied each of the six 6SQuID steps in relation to five areas of adolescent health-related behaviour (substance use, sleep, sexual behaviour, physical activity, and eating behaviour), in order to determine how our review findings could further enhance the development of effective, relevant, and sustainable interventions. This involved incorporating the specific neurological changes that happen during adolescence, such as the alterations that occur in the limbic system and pre-frontal cortex, and their influence on areas such as decision-making and reward processing [7].

The steps outlined in Table 1 were therefore considered in relation to adolescent physiology and health-related behaviour, and the results indicate their application to intervention development.

## Results

### Step 1. Define and understand the problem

The first step in intervention development is clarifying the problem.

We drew upon the existing evidence from our systematic review of adolescent physiology and its influence on health-related behaviour [2], to assist in defining and understanding the issues. We also examined the inter-related influences at social, community, and personal level. We then incorporated these influences into an appropriate diagram for ease of reference.

Our review, which amalgamated findings from 341 papers, detailed seven major conclusions in relation to intervention development. These points help to define and improve understanding of the issues that are of particular salience to adolescent intervention development and implementation. To further clarify how our review findings may help to inform the development of adolescent interventions, an overview of the main findings and conclusions from that report [2] is detailed in Table 2 and falls into three main categories: *decision making*, *reward processing*, and *age and stage*.

**Table 2:** Review findings and implementation considerations

Adolescent physiological factors	Key points from systematic review results	Implementation considerations
Neurological decision-making ability	• When adolescents are in situations that involve a high level of emotion (e.g. times of great excitement), they are less likely to spend time considering the (potentially risky) consequences • In high emotion situations (or so called ‘hot’ emotional contexts) decision-making processes may therefore be different from less charged (‘cold’ or deliberative) situations	• Interventions to support adolescents, and those who care for them, can raise awareness of this likelihood and help adolescents to identify that their ‘gut’, or immediate reaction, in such situations may put them at risk • Knowledge of such variations may help adolescents to differentiate between their decision-making ability in a variety of situations and apply different strategies. For example, gaining the skills to recognise when a rash or unhealthy decision is likely to occur
Reward processing	• Reward processing develops across adolescence	• Interventions to support adolescents should encourage the rewards that healthy behaviours can give (for example being more active and looking/feeling healthier as a result) • In addition, the benefits of saying ‘no’ to unhealthy behaviours can be emphasised (for example refusing cigarettes and having fresher breath, skin, and clothes as a result)
	• In situations where adolescents need to concentrate intensely (for example when playing computer games), they may be more likely to accept an immediate reward without considering the consequences (for example they may be more likely to eat an unhealthy snack and lose their appetite for more nutritious food)	• An understanding of this likelihood may be helpful for both adolescents and those who care for them. In addition, interventions that act as prompts or reminders for adolescents may be of help
Age and stage	• Many new experiences that children encounter as they develop through adolescence can impact on future health (for example, smoking)	• It is important that early interventions, at a younger age, explain the potential consequences (good or bad) in a supportive way. This early approach may help adolescents make healthier choices at a later stage (for example, turning down an offer of a cigarette for the first time)
	• Younger adolescents may be less able to stop acting on impulse, due to the developing (neurological) maturation processes	• Interventions that focus on controlling impulsive action may be better suited to older adolescents
	• Younger adolescents may respond better to interventions that focus on immediate rewards (for example, the immediate benefits of taking exercise, such as glowing skin, feeling energised). In contrast, older adolescents may be better able to see the longer term outcomes and anticipate those benefits (for example, having good muscle tone as a result of regular exercise)	• Interventions that are responsive to and implemented in an *age and stage* appropriate way may be more effective due to their acknowledgement of the physiological maturation process

In addition to the general conclusions from our review, specific points were raised in relation to specific health behaviours. These were incorporated into our examination of singular health behaviours. In addition to this, it is acknowledged that health behaviours are often inter-linked and do not necessarily occur in isolation; however, research studies often focus on, and present evidence from, one behaviour or type of action (e.g. smoking or substance use in general). In the overview of intervention development that follows, we aim to co-ordinate evidence in such a way as to address adolescent issues as a whole whilst still referring to individual areas of concern. It is anticipated that this will assist those who deliver interventions to support adolescents, either relating to individual behaviours (e.g. smoking or safe sex) or more general support (e.g. youth support workers or services).

The 6SQuID steps will be considered in greater depth below, in relation to five areas of adolescent health behaviour (substance use, sleep, sexual behaviour, physical activity, and eating behaviour), before drawing more general intervention conclusions. We will seek to clarify the decision trail at each step in the process of intervention development and improve the likelihood of targeted and appropriate implementation.

Due consideration was given to the salient issues and influential forces across these broad, but potentially interlinked, behaviours. We sought to identify commonalities across behaviours, as well as issues unique to each individual health behaviour.

**Fig. 1:**
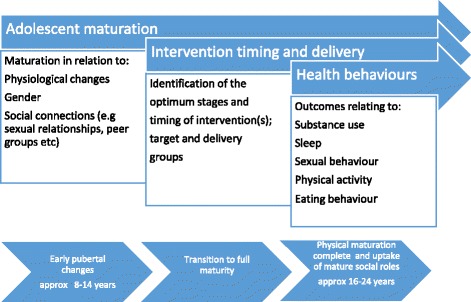
Factors for consideration in relation to adolescent intervention development

From Table 2, it is evident that certain physiological changes during adolescence may predispose adolescents to make unhealthy choices. Knowledge of these factors, in conjunction with associated psycho-social factors, is helpful to inform, focus, and direct interventions (e.g. in respect of timing, gender, or target audiences). Figure 1 illustrates the three main factors that require to be considered in conjunction, as well as the variations in the average timing and stages of adolescence [8].

As illustrated in step 1 and Fig. 1, there are many variables to take into consideration, not least of which are the differences in maturation rates/ages, gender-specific differences, and the optimum stages at which to deliver interventions.

### Step 2. Clarify which causal or contextual factors are malleable and have greatest scope for change

Table 3 identifies factors that have the scope to bring about change, whether this be in relation to their direct influence on adolescents or pathways towards increasing knowledge and understanding that may enhance the likelihood of adolescents making positive health choices. That said, there may be competing and conflicting influences at play, such as school structures and systems, which may be more difficult to alter in the short term. By linking potential outcomes to policy and direction linked to adolescent health, well-being, and education [9, 10], a more persuasive argument for change, albeit in the longer term, may be made. For ease of reference, steps 2 and 4 (identification of how to deliver change) have been combined in Table 3.

**Table 3:** 6SQuID step 2: causal and contextual factors that have scope for change and step 4: how to deliver the change mechanism

6SQuID step	Health behaviours				
	Substance use	Sleep	Sexual behaviour	Physical activity	Eating behaviour
Step 2: clarify which causal or contextual factors are malleable and have greatest scope for change	Positive peer influences and family/social networks can offer a protective effect, and this knowledge can be incorporated into and encouraged within interventions, both at an early stage and as adolescents mature	School schedules may benefit from later start times, if feasible. Ensuring that adolescents themselves, parents, and teachers understand adolescent sleep issues is important; limited late evening screen use and consistent sleep patterns can be promoted	Information may need to be matched to stage of readiness to receive and/or stage of maturity rather than simply age related. Earlier maturation of girls is a factor. Teaching staff and school nurses are well placed to monitor early need for sexual/relationship education, in liaison with parents	Physical activity (PA) programmes may need to be altered to take account of gender and body image issues, especially during periods of rapid physical change in adolescence. PA trainers/teachers are central to implementing changes, such as increasing privacy or altering venues	Information regarding healthy eating needs to be embedded in early education to ensure healthy patterns and understanding predate puberty; school policies regarding meal/snack consumption may be less easy to alter or implement, but ensuring adolescents, teachers, and parents are aware of the nutritional needs of adolescents, and healthy BMI perimeters, may assist. Stress coping strategies can be implemented to help reduce unhealthy eating tendencies linked to anxiety
Step 4: identify how to deliver the change mechanism	Interventions to promote healthy attitudes to substance use may be most effective when they involve peer and social groups throughout adolescence, but certainly starting at an early stage and for those who mature early	Educating adolescents, parents, and teachers about the changing needs of adolescents regarding sleep and sleep patterns may be an effective way of promoting understanding, and good ‘sleep health’	Delivering sex and relationship education according to maturity, stage of readiness to receive, and with gender adaptations may help to target information in the best way and at the optimum time. This may require parental, adolescent, and professional judgement and input	Interventions to promote physical activity in girls need to adapt to take account of changing bodies and self-consciousness and promote the benefits of being and looking healthy. This may be better targeted towards younger adolescents before reduced activity patterns are formed	Encouraging healthy eating behaviour and knowledge can be introduced pre-puberty and emphasised as bodily changes occur during puberty. Links with activity and sleep also need to be emphasised. Stress-coping strategies could be incorporated into life skills sessions
Delivery potential	Youth workers, teachers/teaching assistants, and healthcare workers (e.g. school nurses, GPS) are all well placed to deliver interventions. Parental support and reinforcement of messages may be particularly helpful in areas such as sleep and eating behaviour, especially where parents have had the opportunity to attend information or training sessions and/or contribute to intervention development				

Optimum timing and delivery of interventions may vary. For example, healthy eating, education, and the promotion of positive peer group formation may be more effective during early puberty [2], whereas interventions that seek to influence positive sexual behaviour and physical activity may best be delivered in a gender and maturity stage manner [11, 12].

**Fig. 2:**
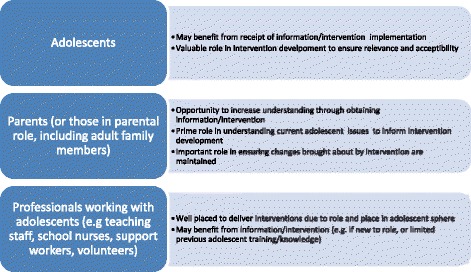
Target populations

With further regard to target populations, these are illustrated in Fig. 2.

As illustrated in Fig. 2, there may be equal benefit for target populations in both receipt of an intervention and intervention development. Ensuring all three groups (adolescents, parental figures, and professionals working with adolescents) are involved in intervention development provides the opportunity for co-production and stakeholder involvement in interventions, as well as being a prospective learning platform.

### Step 3. Identify how to bring about change: the change mechanism

Step 3 acknowledges that linking and underpinning interventions to a relevant theory can assist in identifying how and why change can be influenced [13]. As an example, we have used the Social Learning Theory (SLT) [14]; SLT incorporates several areas that have been identified as pertinent to adolescent health behaviours in steps 1 and 2. These include personal control, support networks, and the environment and context within which support might be required. In addition, SLT takes into account an individual’s self-efficacy (e.g. in terms of confidence, belief, ability, motivation etc.). This may be particularly relevant during adolescence, when increasing independence results in the need to develop greater decision-making and appraisal skills.

With further regard to SLT, there are several salient issues that are especially relevant to the adolescent life stage. For example, with regard to cognitive factors, adolescence is a period during which considerable new knowledge is assimilated and individual attitudes formed [15]. Our review highlighted the fact that it is also a time when impulsive actions may dominate over deliberative decisions, especially during times of heightened emotion. This is something that needs to be understood, both by adolescents and those who care for them, in order to alert them to potential hazardous consequences.

In addition, behavioural factors may influence decision-making in specific respects during this time (e.g. peer pressure). Giving adolescents the skills and confidence to act in uncertain or risky circumstances may have very constructive benefits. For example, being able to decline offers of ‘street’ drugs or refusing to be driven by a peer who has consumed alcohol. Furthermore, environmental factors such as keeping behaviours safe and within the law are in the interests of society as a whole.

Incorporating all these factors into adolescent interventions ensures that a wide range of influential elements and contexts is taken into account and incorporated, to good effect.

### Step 4. Identify how to deliver the change mechanism

Table 3 details where, how, and for whom interventions may be most effective. This includes reference to the type and timing of such input. For example, physiological changes during puberty may cause adolescents to perspire more freely, especially during exercise; for females, this may increase feelings of self-consciousness that are correspondingly associated with changes in physique [16, 17]. It may be that adjustments to the type or venue of activity (e.g. more private settings, provision of shower facilities etc.), or a more discrete dress code that limits feelings of exposure, may act as stimulants to maintain physical activity levels [11].

### Step 5. Test and refine on a small scale

**Fig. 3:**
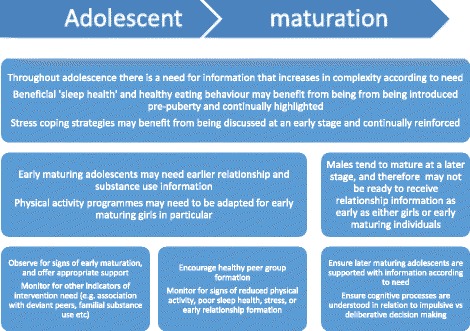
Adolescent intervention delivery

Specific aspects of intervention delivery, as summarised in Table 3, have been incorporated into a maturity-related figure (Fig. 3).

From Fig. 3, it can be seen that intervention delivery may be best achieved in a maturity-related rather than age-related manner. Whilst this process is not prescriptive or definitive, evidence from our review does indicate that certain interventions are better targeted at different maturity levels (e.g. relationship education), as well as for certain at-risk groups (e.g. those with a familial substance use history [18]). In this respect, intervention readiness might best be assessed by a person with good knowledge of the individual in order to gauge optimum receptiveness (e.g. a teacher or health/social professional). Parents are also in a unique position but may be less able to make an objective judgement due to social, religious, or personal beliefs and concerns [19, 20]. Nevertheless, particularly for younger adolescents, parental consent for interventions may still be required, and this needs to be taken into account. Similarly, adolescent/parental spiritual or personal choices need to be respected and preferably known about prior to intervention delivery. Issues relating to intervention delivery could be tested with the target population in a pilot study and appropriate adaptations and refinements made during the intervention development. There may also be a particular need to consider if adaptations are necessary for certain vulnerable or at-risk groups [21].

With regard to the need for increasing complexity of information, adolescent and parental input is vital for assessing acceptability and relevance during intervention development, as detailed previously. For example, information regarding the cognitive processes involved in impulsive versus deliberative decision-making has been placed as a later information need in Fig. 3, due the complex nature of the topic. It may be that certain individuals or groups consider that this information should be delivered earlier. How the intervention is received in practice can be therefore be monitored in a post pilot survey, including process and outcome evaluation [5].

Outcome evaluation should include initial baseline information, where possible, to allow a comparison of pre- and post-intervention scores. Process evaluation can be used to inform interpretation of outcome measures and should include assessments of implementation, mechanisms, and context [22]. Process evaluation is needed to identify implementation problems (as opposed to genuine ineffectiveness) [5] and can also give valuable feedback relating to the relevance and acceptability of content. Such information can be applied in 6SQuID step 6 to alter, define, and justify more widespread intervention delivery.

In relation to resource needs, these will be related to the pilot size, population, and scope. Ideally, the intervention should be trialled on an indicative sample of the target population, in sufficient numbers to be meaningful rather than statistically significant.

If the intervention is delivered across groups (e.g. to a population including professionals, parents, and adolescents), then any evaluation tool would need to identify their sub-group in order to indicate preferences and opinions appropriately.

### Pilot study example

Using the above principles, a pilot study that targets teaching assistants is used to illustrate a training intervention. Teaching assistants do not generally have any formal teaching qualifications but play a pivotal role in the support of pupils. Their assistance may be vital to pupils, especially in challenging situations where behavioural or learning difficulties may be present [23, 24]. Training that is specific to adolescent development has the potential to improve knowledge and benefit practice [25], particularly for those without prior training or new to the role. This is summarised in Table 4.

**Table 4:** 6SQuID step 6: pilot intervention considerations

Pilot considerations	Details
Study approvals	Applications made to, and approvals sought from, relevant ethics committee(s) and education board(s)
Sampling	Local schools identified. Head/lead teachers provided with information about the study, whom it would involve and how it would be carried out (e.g. details of time commitment, venue etc.)
Population	Teaching assistants do not generally have any formal teaching qualifications but may be vital to the support of pupils, especially in challenging situations. Training that is specific to adolescent development may be of benefit, particularly for those new to the role
Recruitment	Head teachers, who are agreeable to study conduct at their school, would be asked to pass study details to their teaching assistants. Those willing to take part would be asked to consider the study information and sign a consent form, which would then be given to the researchers. A suitable time and venue for the training session would be agreed with the head teacher and teaching assistants to minimise disruption. Participants would be asked to complete a short pre-intervention questionnaire to assist evaluation (e.g. assessing level of existing knowledge and understanding)
Intervention	Aim: to improve knowledge and confidence in dealing with adolescents, through increased understanding of adolescent development. Objective: to deliver a 30-min slide show detailing key issues in adolescent development and behaviour-influencing factors. The intervention would include details about why and when support might be needed, identification of those who might be more in need of support (e.g. at risk groups), what type of support teaching assistants can provide, and when to alert or refer to more senior staff or other professionals (including consideration of confidentiality issues). The slide show information would be complied into a written booklet for future reference. The intervention would be concluded with a question and answer session, as well as general discussion. Permission to record and use this part of the intervention would be sought, both as part of study permissions and from individual participants. Brief post-intervention questionnaire given/completed. Total intervention time: approx. 1 h
Outcome measures	Participant levels of understanding and knowledge Acceptability and relevance of intervention Influence on future practice (e.g. confidence in dealing with adolescent issues)
Resource requirements	Study base: office for study team to operate from, including access to IT/PC, stationery, telephones etc. Study team personnel: to develop study protocol, including evaluation tools, execute approval submissions, recruit consenting participants, deliver and evaluate intervention, write/deliver study report etc. Venue: appropriate intervention venue to be sourced and agreed, including consideration of accessibility, refreshment, and toilet facilities. Power supply and appropriate IT facilities for intervention delivery. Other resource requirements: production and supply of study information sheets, intervention booklets, and questionnaires. Transport costs and connections to intervention venues
Evaluation	Outcome evaluation: pre- and post-intervention ratings of levels of understanding, knowledge, confidence, and influence on future practice (from questionnaires) Process evaluation: post-intervention questionnaire/interview (venue, content, delivery method etc.)

The example detailed in Table 4 gives a suggested outline of intervention delivery, including process considerations and evaluation. These details are adaptable to other participant groups, according to level of requirement and complexity. Evaluation will provide specific valuable data to inform broader implementation, once any necessary adaptations have been made.

### Step 6. Collect sufficient evidence of effectiveness to justify rigorous implementation or evaluation

Step 6 allows for a full-scale intervention to be developed and for data to be collected and examined in order to ensure that the intervention is working as intended, using the knowledge gained from the previous five steps. Each step can therefore be seen to build on the previous steps, in a manner that incorporates, rather than duplicates, information. This illustration of the pathways through which the 6SQuID process works therefore demonstrates its usefulness in intervention development. Whilst strict standardisation of intervention delivery may be inappropriate across diverse groups and settings, it is important that evaluation during and following step 6 includes assessment of implementation fidelity. This will ensure that any necessary adaptations to fit differing contexts, whilst allowing for flexibility, do not alter or undermine the active components and delivery of the intervention [22].

## Discussion

This paper has examined, in depth, the process of intervention development using a defined method and process. The progression through each step has been clearly illustrated, and a sample intervention has been given for further clarity. An acknowledged limitation is the fact that an intervention was not delivered in practice. However, there may be further opportunity to do this in the future, now that the route for accomplishing it has been specified.

Further limitations relate to the potential arbitrary cut-offs in the process of intervention development, and the linear progression, suggested by the 6SQuID framework. However, in practice this is often an iterative process, and intervention developers will return to earlier steps before reaching step 6, as we did. If each of these six steps is carefully addressed in the design of interventions, better use will be made of scarce public resources by avoiding the costly evaluation, or implementation, of unpromising interventions [4].

Intervention development can be a complicated process and one that can prove futile if interventions are not devised and implemented appropriately [5]. Tools such as 6SQuID, that aim to assist in the development of interventions, ensure that appropriate consideration is given to all necessary elements during the process. Adolescence is itself a complex journey, described as ‘a phase of life beginning in biology and ending in society’ [26]. Influencing factors, such as the physiology of biological development and the psychosocial contexts in which interventions may take place, need to be fully understood and integrated into interactions with adolescents, and those who care for them, to have the best chance of being effective.

With further regard to intervention delivery, there are several ways in which the information presented here may assist adolescents and those who care for them. These include:

- Contributing to existing interventions for adolescents, to ensure delivery is acceptable and relevant to need
- Directly informing new intervention proposals for delivery to adolescents
- To help advise professionals about factors they need to be aware of in their interactions with adolescents, whether in informal or formal contexts
- Informing policy makers with an interest in child and adolescent development
- To assist parents (or those acting in a parental role) to understand the varying needs of their adolescents
- Directly to inform adolescents about their maturation and changing circumstances

## Conclusions

This paper has given details of how the 6SQuID framework can be used for intervention development, using specific research findings, across a variety of health behaviours. By incorporating relevant aspects and features of adolescence, this example will be of interest to a wide variety of people who are involved with the delivery of interventions for, and with, adolescents. Furthermore, the example provides details of how to operationalise 6SQuID in practical terms that are transferrable to other populations and situations. In this respect, we anticipate that this illustrative case may be of use in the design, development, and implementation of a wide variety of interventions.
